# Effects of arousal and valence on standing kinematics and kinetics in young men: An approach using a motion capture system

**DOI:** 10.14814/phy2.70559

**Published:** 2025-09-16

**Authors:** Ryogo Takahashi, Naotsugu Kaneko, Kimitaka Nakazawa

**Affiliations:** ^1^ Department of Life Sciences, Graduate School of Arts and Sciences The University of Tokyo Tokyo Japan; ^2^ Research Fellow of the Japan Society for the Promotion of Science (JSPS) Tokyo Japan

**Keywords:** autonomic nervous system, biomechanics, breathing, emotion, heart rate, postural control

## Abstract

The emotional impact on body kinematics and kinetics during quiet standing was investigated using a motion capture system. Twenty‐two participants stood on a force plate while viewing affective pictures for 72 s. The task was repeated six times under different picture conditions categorized by arousal (High and Low) and valence (Unpleasant, Neutral, and Pleasant). We analyzed variabilities of the center of mass, lower‐limb joint angles (ankle, knee, and hip), and joint torques. Additionally, breathing parameters, including tidal volume, breathing rate, and minute ventilation, were estimated based on abdominal movements. The results showed that valence significantly influenced postural kinematics and kinetics, with unpleasant emotions reducing variabilities of the center of mass, ankle angle, and ankle torque, implying ankle‐driven postural control. Additionally, pleasant emotions increased breathing rate. On the other hand, high arousal states increased tidal volume and minute ventilation compared to low arousal states. These findings suggest that emotional states manifest as changes in body kinematics and kinetics during quiet standing, extending upon traditional center of pressure (COP)‐based reports. Our study provides new insights into emotional effects on physical responses and highlights the utility of standing parameters measured by motion capture systems for emotional detection.

## Graphical abstract

Both arousal and valence influence body kinematics and kinetics during quiet standing. These parameters could serve as valuable tools for emotional detection.

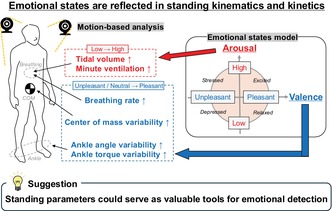

## 1 INTRODUCTION

Emotion can elicit physiological and behavioral responses, allowing individuals to react to affective environmental events (Darwin, 1872). To date, emotional effects on physiological and behavioral responses have been widely examined through Russell's emotional model, which is composed of arousal (High/Low) and valence (Pleasant/Unpleasant) (Lang et al., 2005; Russell, 1980; Wang et al., 2022). While numerous studies have documented the effects of arousal and valence on physiological variables (e.g., cardiac activity and electrodermal responses (Kreibig, 2010)), the exploration of behavioral indicators (e.g., body kinematics and kinetics) reflecting these emotional states still holds potential for further investigation. One of the behavioral responses sensitive to emotions is the center of pressure (COP) during standing posture measured by a force plate (Monéger et al., 2025, for a review). Because COP trajectory closely resembles that of the center of mass (COM) during quiet standing (Winter et al., 1998), COP is widely used as an indicator of body sway (Paillard & Noé, 2015). More specifically, from the perspective of inverse dynamics, the COP during quiet standing is almost proportional to the ankle torque (Masani et al., 2003; Winter et al., 1998); therefore, emotions have impacts on standing postural control by adjusting the ankle torque. Specifically, some studies have shown valence effects on the COP, characterized by the reduction of COP variability (e.g., standard deviation) or increase of COP frequency (e.g., mean power frequency) (Azevedo et al., 2005; Ciria et al., 2017; Facchinetti et al., 2006; Roelofs et al., 2010; Takahashi, Kaneko, Yokoyama, et al., 2024), and other studies have reported that aroused emotions induce similar COP changes (Horslen & Carpenter, 2011; Kordts‐Freudinger et al., 2017; Mouras et al., 2015).

Nevertheless, a major limitation of using the COP is that it provides information about the ankle's contribution to postural control in terms of the inverse dynamics (Masani et al., 2003; Winter et al., 1998). Although it was traditionally believed that quiet standing, without any perturbation, is mainly regulated by ankle torque (Horak & Nashner, 1986), recent works have revealed significant roles of the other lower‐limb joints (Shanbhag et al., 2023), particularly the hip joint, in quiet standing control (Aramaki et al., 2001; Sasagawa et al., 2009, 2014). For example, the ankle and hip joints coordinate with each other to minimize the COM acceleration during quiet standing (Sasagawa et al., 2014). Therefore, it is possible that the hip joint would be involved in postural modulation influenced by an emotional factor.

In addition to the multi‐joint contribution to quiet standing control, thoracic and abdominal movements associated with breathing are also deeply related to quiet standing (Bouisset & Duchêne, 1994; Hodges et al., 2002). These movements are closely coordinated (Zoumot et al., 2015). Notably, both thoracic and abdominal breathing generate body sway, potentially destabilizing postural balance. In other words, thoracic and abdominal breathing movements are the visible kinematic feature during quiet standing. Besides, it has been well established that emotional states influence respiratory parameters (Boiten, 1998; Gomez et al., 2004, 2005, 2008; Gomez & Danuser, 2004; Gomez, Filippou, et al., 2016). In particular, minute ventilation (e.g., L/min), estimated by multiplication of tidal volume (e.g., L) and breathing rate (e.g., bpm), has been well established as a reliable parameter of arousal states, with high arousal emotions increasing minute ventilation (Boiten, 1998; Gomez et al., 2004, 2005, 2008; Gomez & Danuser, 2004; Gomez, Filippou, et al., 2016). Moreover, it has been shown that cognitive load, which elevates arousal levels, influences respiratory parameters derived from abdominal breathing movements during quiet standing (Hagio et al., 2018). Thus, abdominal breathing movements may capture emotional modulation during quiet standing.

A motion capture system is a powerful tool for assessing whole‐body dynamics and has the potential to visualize emotional states, much like a force plate. In the present study, we aimed to investigate the effects of arousal and valence on body kinematics and kinetics during quiet standing using a motion capture system, thereby extending previous findings based on COP measures (Horslen & Carpenter, 2011; Takahashi, Kaneko, Yokoyama, et al., 2024). Considering the continuous body sway as an inherent property of standing posture, we focused on (1) variabilities of the COM, lower‐limb joint angles, and torques as postural kinematic and kinetic parameters. Additionally, we focused on (2) respiratory parameters estimated by abdominal breathing movements. Based on previous findings that valence influences COP variability (i.e., reduction under unpleasant emotions) (Takahashi, Kaneko, Yokoyama, et al., 2024), we hypothesized that variabilities of the COM, ankle and hip angles, and torques would be reduced in unpleasant emotions compared to neutral and pleasant emotions (Hypothesis 1: H‐1). Additionally, we expected that high arousal emotions would increase the minute ventilation (H‐2).

## 2 METHODS

### 2.1 Participants

Prior to the experiment, we conducted a power analysis to determine the required sample size using G*Power (ver. 3.1). Specifically, we referred to a previous study that reported effects of arousal and valence on COP variables during quiet standing (Horslen & Carpenter, 2011) and used the effect size of the COP variable (mean power frequency) affected by emotional states to calculate the sample size (effect size ($\eta_{p}^{2}$): 0.151; *α* level: 0.05; power (1‐*β* error probability): 0.95). As a result, the calculated necessary number of participants was twenty‐two. According to the power analysis result, 22 healthy male volunteers were recruited from the graduate and undergraduate students at The University of Tokyo. The mean age, weight, and height of the participants with standard deviation (SD) were 24.24 ± 3.4 years, 67.1 ± 11.6 kg, and 173.0 ± 5.5 cm, respectively. All participants provided written informed consent to participate in the present study, and the experimental procedures were approved by the Institutional Review Board (IRB) of the University of Tokyo (Number: 792). This study was performed in accordance with the Declaration of Helsinki (1964). Before the experiment, the participants answered 21 questions on the Beck Depression Inventory‐II (Beck et al., 1996) to assess their degree of depression.

### 2.2 Affective stimuli and conditions

The International Affective Picture System (IAPS) (Lang et al., 2005) was used for affective intervention. Self‐Assessment Manikin (SAM) (Bradley & Lang, 1994) values were attached to each picture as quantified arousal and valence values. SAM values ranged from 1 to 9: low values indicated low‐arousal or unpleasant valence, and high values indicated high‐arousal or pleasant valence. Twelve pictures were selected for the following six conditions comprising two arousal conditions (High and Low) and three valence conditions (Pleasant, Neutral, and Unpleasant): (1) High‐Pleasant, (2) High‐Neutral, (3) High‐Unpleasant, (4) Low‐Pleasant, (5) Low‐Neutral, and (6) Low‐Unpleasant. Appendix provides the affective pictures used for each condition.

### 2.3 Data collection

Participants were asked to wear tight‐fitting clothing to ensure accurate placement of motion capture markers. Electrocardiogram (ECG) data were recorded by attaching two bipolar Ag/AgCl surface electrodes (Vitrode F‐150S, Nihon Kohden, Tokyo, Japan) to the skin. Before the electrodes were applied, the skin was cleaned with alcohol to reduce impedance. A ground electrode was placed immediately below the right patella. ECG data were recorded using a lead II pattern with two electrodes. The ECG signals were band‐pass filtered (0.08–1000 Hz) and amplified (×1000) using a multichannel amplifier (MEG‐6108, Nihon Kohden, Tokyo, Japan). All ECG data were recorded at a sampling rate of 1000 Hz using an analog‐to‐digital (A/D) converter (Powerlab/16SP, AD Instruments, Castle Hill, Australia).

**FIGURE 1:**
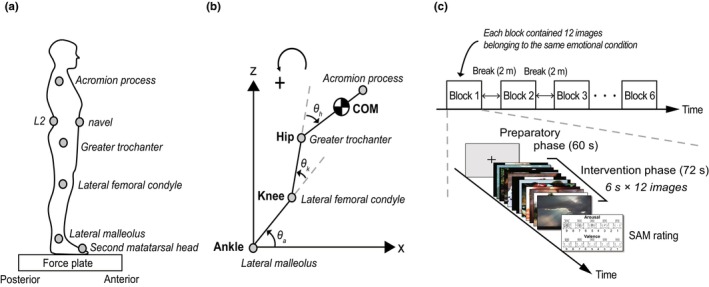
(a) Reflective marker placement attached on the body. The gray circles indicate maker locations. (b) Stick diagram of the quiet standing posture modeled as the three‐segment (leg–thigh–HAT) in the sagittal plane. Counterclockwise is defined as positive. The body is exaggeratedly tilted forward for ease of viewing. (c) Schematic of the entire experimental timeline and individual blocks. The experiment consisted of six blocks, with 2‐min breaks between each block. Each block started with a 60 s preparatory phase followed by a 72 s intervention phase. In the intervention phase, 12 images were displayed for 6 s each. Finally, the participants rated their emotional states using the SAM value.

Ground reaction forces and moments were recorded using a force plate embedded in a split‐belt treadmill at a sampling rate of 1000 Hz (Bertec, Columbus, OH, USA). Kinematic data were obtained using an optical motion capture system (OptiTrack: V100R2, NaturalPoint, Corvallis, OR, USA) with nine infrared cameras at a sampling rate of 100 Hz. Seven reflective markers (5 mm diameter) were placed on specific body parts: the second metatarsal head, lateral malleolus, lateral femoral condyle, greater trochanter, acromion process on the right side of the body, the navel, and the second lumbar vertebra (L2) (Figure 1a). Among these, five reflective markers placed on the second metatarsal head, lateral malleolus, lateral femoral condyle, greater trochanter, and acromion process were used to obtain the motion of the leg, thigh, and head–arms–trunk (HAT) segments (Figure 1b). It has been reported that these three segments are essential at least for modeling quiet standing (Yamamoto et al., 2015). The other two markers (the navel and L2) were used to detect abdominal breathing movements (Hagio et al., 2018).

### 2.4 Procedures

Participants stood on a force plate with their feet shoulder‐width apart and arms relaxed along the trunk. A 24‐inch monitor was placed 1 m in front of the participants, and its height was adjusted to the eye line. The experiment consisted of six blocks, and each block started with a 60 s preparatory phase followed by a 72 s intervention phase (Figure 1c). A fixation cross was displayed in the preparatory phase, and in the intervention phase, 12 affective pictures belonging to the same emotional condition were displayed. Affective pictures from different emotional conditions were not mixed within a block. Each affective picture was displayed successively for 6 s without any interval. The experimental block order was randomized across participants. Two‐minute breaks were set between the blocks. After each block, the participants rated subjective arousal and valence scores for each affective picture using SAM values closest to the face of the manikin.

### 2.5 Data analysis

In line with a previous study, two participants with depression scores >17 were excluded from the analysis (Lebert et al., 2020). Additionally, two other participants who reported voluntary movements and fatigue were excluded from the analysis. Ultimately, eighteen participants were included in the analysis. All signals were analyzed using a custom‐written script in MATLAB (2024a, MathWorks Inc.). Signals recorded only in the intervention phase were analyzed because the preparatory phase was set to stabilize the standing posture.

#### 2.5.1 Heart rate

All ECG data were low‐pass filtered at 20 Hz using a second‐order Butterworth filter. Next, R‐waves were detected in the time‐series data of the ECG to obtain the R‐R interval. The heart rate was then calculated using the mean R‐R interval during the intervention phase. The heart rate is well‐established objective indicator of valence states (Bradley et al., 2001), and it typically decreases while viewing unpleasant images.

#### 2.5.2 COP variables

The force plate signals were low‐pass filtered at 5 Hz using a second‐order Butterworth filter, and the COP in the anterior–posterior (AP) direction was calculated. The following three parameters were calculated from the COP for each condition: standard deviation (SD‐COP) as the COP variability, mean power frequency (MPF‐COP) as the COP frequency, and mean velocity (MV‐COP) as the COP velocity. For the preprocessing step in calculating the MPF‐COP, the 72 s data in each block were divided into 16 segments (50% overlapping) with 8192 data points. A 13‐bit fast Fourier transform was applied to each segment after applying a Hanning window to each segment. The frequency resolution was approximately 0.12 Hz (1000 Hz / 8192 data points). Subsequently, the auto‐power spectrum density (PSD) of the COP displacement was calculated for each segment and then averaged across segments. Then, MPF‐COP was calculated using Equation (1):

(1) $$\mathrm{MPF}\;\left[\mathrm{Hz}\right]=\frac{\sum f\cdot P\left(f\right)}{\sum P\left(f\right)}$$

where f is the frequency of the COP signal and P is the PSD at the frequency.

We previously confirmed that the COP variables—identical to those calculated in the present study—showed minimal differences between the force plate embedded in the treadmill and a standard force plate (Takahashi, Kaneko, Yokoyama, et al., 2024). Therefore, the use of the treadmill unlikely affected the results in the present study.

#### 2.5.3 Postural kinematics and kinetics

As preprocessing, the coordinates of five reflective markers (the second metatarsal head, lateral malleolus, lateral femoral condyle, greater trochanter, and acromion process) were low‐pass filtered at 2 Hz using a second‐order Butterworth filter to remove artifacts. Next, the joint angles of the ankle ($\theta_{a}$), knee ($\theta_{k}$), and hip ($\theta_{h}$) were calculated based on the coordinates of the five reflective markers. Subsequently, the COM in the AP direction $\left(x_{\mathrm{COM}}\right)$ was computed using Equation (2):

(2) $$x_{\mathrm{COM}}=k_{1}\theta_{a}+k_{2}\theta_{k}+k_{3}\theta_{h}$$

where $k_{1}$, $k_{2}$, and $k_{3}$ are constants determined from individual anthropometric measurement and standard anthropometric data (Winter, 2009).

Joint torques of the ankle, knee, and hip were estimated using a top‐down inverse dynamics approach based on the leg–thigh–HAT model. Specifically, the Newton–Euler equations were sequentially solved for each segment—HAT, thigh, and leg—in that order, to compute joint torques and forces using the following matrix formulation:

(3) $$\begin{array}{c} A\cdot x=b \end{array}$$

where $x={\left[F_{x},F_{z},\tau \;\right]}^{T}$ represents the unknown distal joint forces and torque, and the matrix A and vector b were constructed as:

(4) $$\begin{array}{c} A=\left[\begin{array}{ccc} 1 & 0 & 0\\ 0 & 1 & 0\\ -r_{z} & r_{x} & 1 \end{array}\right] \end{array}$$

(5) $$\begin{array}{c} b=\left[\begin{array}{c} m\cdot a_{x}+F_{\text{prox},x}\\ m\cdot a_{z}+F_{\text{prox},z}-m\cdot g\\ I\cdot \alpha -r_{\text{prox},z}F_{\text{prox},x}+r_{\text{prox},x}F_{\text{prox},z}+\tau_{\text{prox}} \end{array}\right] \end{array}$$

where m is the segment mass, I is the segment's moment of inertia, $\left(a_{x},a_{z}\right)$ is the acceleration of the segment's center of mass, g is the gravitational acceleration, α is the angular acceleration, and $\left(r_{x},r_{z}\right)$ is the vector from the proximal joint to the segment's center of mass. $F_{\text{prox}}$ and $\tau_{\text{prox}}$ represent the distal joint forces and torque calculated in the previous step.

As postural kinematic and kinetic parameters, the following variability measures were calculated: (1) standard deviation of the COM (SD‐COM), (2) standard deviation of joint angles (SD‐angle‐ankle, SD‐angle‐knee, and SD‐angle‐hip), and (3) standard deviation of joint torques (SD‐torque‐ankle, SD‐torque‐knee, and SD‐torque‐hip). Since COM and joint angle signals primarily reflect low‐frequency fluctuations, and torque signals derived from inverse dynamics may contain high‐frequency noise, we selected SD as a robust and interpretable measure of variability across all parameters.

#### 2.5.4 Breathing variables

Abdominal breathing movements were estimated by the sagittal distance between two reflective markers placed on the navel and L2 (the abdominal displacement) (Hagio et al., 2018). To extract the pure abdominal breathing movements, the abdominal displacement was band‐pass filtered at 0.1 to 0.4 Hz using a second‐order Butterworth filter (Iqbal et al., 2022) Subsequently, the following three breathing variables were calculated (Romei et al., 2010): (1) tidal volume, (2) breathing rate, and (3) minute ventilation. Tidal volume was quantified as the mean peak‐to‐peak amplitude of the abdominal displacement (Reyes et al., 2017). Breathing rate was calculated by detecting the durations between successive positive peaks of the abdominal displacement during the task, transforming them to the number of breathings in a minute, and averaging them. Minute ventilation was defined by the multiplication of the tidal volume and breathing rate (i.e., tidal volume × breathing rate).

### 2.6 Statistical analysis

All statistical comparisons were performed using R software package (ver. 4.1.2).

#### 2.6.1 Comparison among conditions

Shapiro–Wilk tests were conducted to confirm the normal distribution of the parameters. Because the parameters showed a non‐normal distribution, all data were transformed using the aligned rank transformation procedure in the R package “ARTool (Kay et al., 2021).” The aligned rank transformation allows non‐normally distributed data to be applied to the analysis of variance (ANOVA) of the parametric method (Wobbrock et al., 2011). Next, a two‐way repeated measures ANOVA with arousal (High and Low) and valence (Unpleasant, Neutral, and Pleasant) as within‐subject factors was performed on all the variables. A post hoc contrast test in ARTool was conducted when a significant main effect of arousal or valence was observed (Elkin et al., 2021). When a significant interaction was observed, simple main effects of arousal or valence in each valence and arousal level were examined, and the post hoc contrast test was conducted in the case of a significant simple main effect of valence in each arousal level. All *p* values were corrected using false discovery rate correction (Benjamini–Hochberg method). The effect sizes for the ANOVA and post‐hoc tests were calculated as partial eta squared ($\eta_{p}^{2}$) and Cohen's d (*d*), respectively. The significance level for all tests was set to *p* <0.05.

#### 2.6.2 Correlation analysis

To examine whether changes in COM variability (i.e., SD‐COM) as an indicator of postural sway between conditions are associated with changes in other standing kinematic and kinetic parameters, we calculated Kendall's correlation coefficient (*τ*) between the change ratio of SD‐COM and those of the parameters described in Sections 2.5.2, 2.5.3, and 2.5.4, for the condition pairs that showed significant differences in SD‐COM. All *p* values obtained from the correlation analysis were corrected using false discovery rate correction per variable pair, with the significance threshold set at *p* <0.05.

## 3 RESULTS

To assess whether the sample size of 18 participants was statistically appropriate, we conducted a post hoc power analysis based on the significant main effect of valence on MPF‐COP (see Section 3.3 COP variables). This analysis indicated a power of 0.792 (effect size: $\eta_{p}^{2}$ = 0.309; *α* level = 0.05), which is close to the widely accepted threshold of 0.80 (Cohen, 1998).

### 3.1 Subjective emotional ratings

**FIGURE 2:**
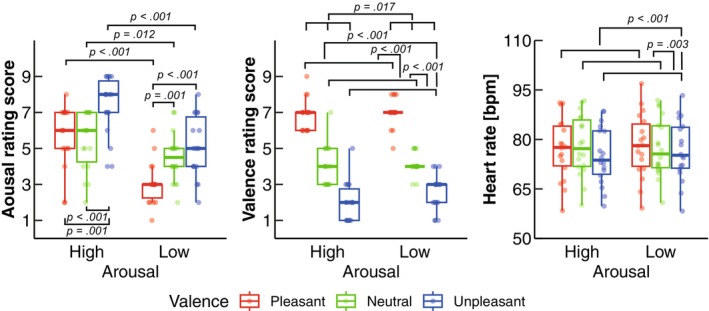
Group data for the emotional rating score and heart rate. The lines in the box plots indicate median values, and the ends of the boxes represent the 25th and 75th percentiles. The dots represent individual data points. The significant *p* values (*p* < 0.05) are indicated.

Table 1 summarizes the statistical results of two‐way ART‐ANOVA (two arousal × three valence) while Table 2 shows results of post hoc tests following significant main effect of valence or interaction. The two‐way ART‐ANOVA revealed a significant interaction on the arousal rating (*p* = 0.015; Table 1; Figure 2). Significant simple main effects of arousal on the arousal rating were observed in each valence level, and the rating scores were higher in High than in Low (Unpleasant: *p* < 0.001; Neutral: *p* = 0.012; Pleasant: *p* < 0.001; Table 2; Figure 2). In addition, significant simple main effects of valence on the arousal rating were observed in each arousal level. When arousal was High, the arousal rating was higher in Unpleasant than in Neutral (*p* < 0.001) and Pleasant (*p* = 0.001; Table 2; Figure 2). When arousal was Low, the arousal rating was lower in Pleasant than in Unpleasant (*p* < 0.001) and Neutral (*p* = 0.001; Table 2; Figure 2). The two‐way ART‐ANOVA showed significant main effects of arousal (*p* = 0.017) and valence (*p* < 0.001) on valence rating (Table 1; Figure 2). For the main effect of arousal, the rating score was higher in Low than in High (Figure 2). In addition, post hoc contrast tests showed higher rating scores in Pleasant than in Unpleasant (*p* < 0.001) and Neutral (*p* < 0.001), and in Neutral than in Unpleasant (*p* < 0.001; Table 2; Figure 2).

**TABLE 1:** Summary of the two‐way ART‐ANOVA (arousal × valence) of the variables.

	Arousal main effect			Valence main effect			Arousal × Valence interaction		
	*F*_(1,85)_	*p* Value	$\eta_{p}^{2}$	*F*_(2,85)_	*p* Value	$\eta_{p}^{2}$	*F*_(2,85)_	*p* Value	$\eta_{p}^{2}$
Subjective emotional ratings									
Arousal rating	57.9	**< 0.001**	0.405	15.7	**< 0.001**	0.269	4.38	**0.015**	0.093
Valence rating	5.92	**0.017**	0.065	246	**< 0.001**	0.853	1.42	0.247	0.032
Heart rate									
Heart rate	1.16	0.285	0.013	7.68	**< 0.001**	0.153	2.63	0.077	0.058
COP variables									
SD‐COP	0.314	0.576	0.003	2.91	0.059	0.064	0.044	0.956	0.001
MPF‐COP	0.208	0.649	0.002	4.06	**0.020**	0.087	0.085	0.917	0.002
MV‐COP	0.734	0.393	0.008	0.385	0.681	0.008	3.21	**0.045**	0.070
Postural kinematics and kinetics									
SD‐COM	0.408	0.524	0.005	4.85	**0.010**	0.102	0.362	0.697	0.008
SD‐ankle‐angle	0.122	0.726	0.001	4.65	**0.012**	0.099	0.513	0.600	0.012
SD‐knee‐angle	1.59	0.211	0.018	0.332	0.718	0.008	0.877	0.419	0.020
SD‐hip‐angle	0.716	0.400	0.001	1.90	0.155	0.043	0.798	0.454	0.018
SD‐ankle‐torque	0.650	0.422	0.008	3.73	**0.027**	0.081	0.261	0.539	0.014
SD‐knee‐torque	0.774	0.381	0.009	1.54	0.221	0.035	1.68	0.192	0.038
SD‐hip‐torque	0.037	0.848	0.000	1.31	0.274	0.030	1.17	0.314	0.027
Breathing variables									
Tidal volume	7.12	**0.009**	0.077	0.021	0.979	0.000	0.571	0.567	0.013
Breathing rate	2.10	0.151	0.024	3.69	**0.029**	0.079	0.349	0.706	0.008
Minute ventilation	8.65	**0.004**	0.092	0.267	0.766	0.006	0.796	0.454	0.018

*Note*: Significant *p* values (<0.05) are in boldface.

**TABLE 2:** Summary of the post hoc tests following significant main effects or interactions identified by the two‐way ART‐ANOVA (arousal × valence).

Post hoc contrast tests following significant main effect of valence									
	Pleasant versus Neutral			Neutral versus Unpleasant			Unpleasant versus Pleasant		
	*t*_(85)_	*p* Value	*d*	*t*_(85)_	*p* Value	*d*	*t*_(85)_	*p* Value	*d*
Valence rating	12.0	**<0.001**	2.65	10.2	**<0.001**	2.24	22.2	**<0.001**	4.89
Heart rate	0.373	0.709	0.025	3.19	**0.003**	0.216	3.57	**0.001**	0.241
MPF‐COP	1.34	0.182	0.176	1.50	0.182	0.197	2.85	**0.016**	0.373
SD‐COM	0.807	0.421	0.112	2.20	**0.045**	0.306	3.01	**0.010**	0.418
SD‐ankle‐angle	0.754	0.452	0.169	2.18	**0.047**	0.284	2.94	**0.012**	0.453
SD‐ankle‐torque	0.905	0.367	0.098	1.78	0.118	0.217	2.69	**0.026**	0.315
Breathing rate	2.69	**0.025**	0.244	1.65	0.153	0.159	1.04	0.301	0.085

Post hoc tests following significant interaction									
*Post hoc contrast test* (*arousal*) *in each valence level*									
	Pleasant			Neutral			Unpleasant		
	*t*_(17)_	*p* Value	*d*	*t*_(17)_	*p* Value	*d*	*t*_(17)_	*p* Value	*d*
Arousal rating	5.73	**<0.001**	1.71	2.79	**0.012**	1.37	*4.97*	**<0.001**	1.37
MV‐COP	2.09	0.051	0.284	1.45	0.164	0.241	1.28	0.215	0.252

*One‐way ART‐ANOVA* (*valence*) *in each arousal level*						
	High			Low		
	*F*_(2,34)_	*p* Value	$\eta_{p}^{2}$	*F*_(2,34)_	*p* Value	$\eta_{p}^{2}$
Arousal rating	11.1	**<0.001**	0.394	12.0	**< 0.001**	0.413
MV‐COP	1.93	0.159	0.102	1.97	0.154	0.103

*Post hoc contrast test following significant simple main effect of valence in High*									
	Pleasant versus Neutral			Neutral versus Unpleasant			Unpleasant versus Pleasant		
	*t*_(34)_	*p* Value	*d*	*t*_(34)_	*p* Value	*d*	*t*_(34)_	*p* Value	*d*
Arousal rating	0.821	0.417	0.239	4.42	**<0.001**	1.29	3.60	**0.001**	1.05

*Post hoc contrast test following significant simple main effect of valence in Low*									
	Pleasant versus Neutral			Neutral versus Unpleasant			Unpleasant versus Pleasant		
	*t*_(34)_	*p* Value	*d*	*t*_(34)_	*p* Value	*d*	*t*_(34)_	*p* Value	*d*
Arousal rating	3.58	**0.001**	1.14	1.10	0.280	0.350	4.68	**<0.001**	1.49

*Note*: When a significant main effect of valence was detected, post hoc contrast tests were conducted among the three valence levels (Pleasant, Neutral, and Unpleasant). When a significant interaction was detected, a one‐way ART‐ANOVA (for arousal) was first performed within each valence level to detect the simple main effect of arousal, followed by post hoc contrast tests where appropriate. Significant *p* values (<0.05) are in boldface.

### 3.2 Heart rate

Two‐way ART‐ANOVA revealed a significant main effect of valence on the heart rate (*p* < 0.001; Table 1). Post hoc contrast tests revealed a lower heart rate in Unpleasant than in Neutral (*p* = 0.003) and Pleasant (*p* = 0.001; Table 2; Figure 2).

### 3.3 COP variables

**FIGURE 3:**
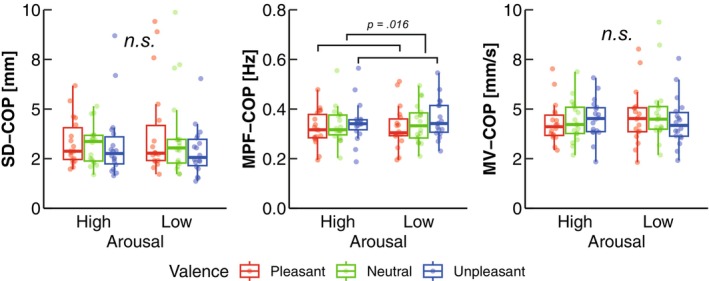
Group data for the COP variables. The lines in the box plots indicate median values, and the ends of the boxes represent the 25th and 75th percentiles. The dots represent individual data points. The significant *p* value (*p* < 0.05) is indicated. n.s. shows nonsignificant *p* value.

Two‐way ART‐ANOVA showed a significant main effect of valence on MPF‐COP (*p* = 0.020; Table 1; Figure 3). Post hoc contrast tests revealed that MPF‐COP was significantly higher in Unpleasant than in Pleasant (*p* = 0.016; Table 2; Figure 3). The two‐way ART‐ANOVA showed a significant interaction on MV‐COP (*p* = 0.045; Table 1; Figure 3). However, no significant simple main effect of arousal was observed in each arousal level (All *p* > 0.05; Table 2; Figure 3). Similarly, no significant difference between two arousal levels was observed in each valence level (All *p* > 0.05; Table 2; Figure 3). The two‐way ART‐ANOVA did not show any significant main effect or interaction on SD‐COP (Table 1; Figure 3).

### 3.4 Postural kinematics and kinetics

**FIGURE 4:**
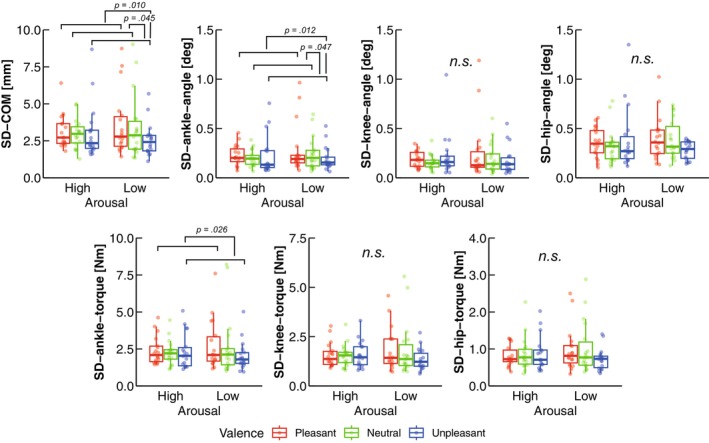
Group data for the postural kinematic and kinetic variables. The lines in the box plots indicate median values, and the ends of the boxes represent the 25th and 75th percentiles. The dots represent individual data points. The significant *p* values (*p* < 0.05) are indicated. n.s. shows nonsignificant *p* value.

The two‐way ART‐ANOVA showed a significant main effect of valence on SD‐COM (*p* = 0.010; Table 1; Figure 4). Post hoc contrast tests confirmed that SD‐COM was significantly lower in Unpleasant than in Neutral (*p* = 0.045) and Pleasant (*p* = 0.010; Table 2; Figure 4). Regarding the joint angular variability, the two‐way ART‐ANOVA showed a significant main effect of valence on SD‐ankle‐angle (*p* = 0.012; Table 1; Figure 4). The post hoc contrast tests confirmed that SD‐angle‐ankle was significantly lower in Unpleasant than in Neutral (*p* = 0.047) and Pleasant (*p* = 0.012; Table 2; Figure 4). In contrast, no significant main effect or interaction on SD‐knee‐angle or SD‐hip‐angle was found (All *p* > 0.05; Table 1; Figure 4).

The two‐way ART‐ANOVA showed a significant main effect of valence on SD‐ankle‐torque (*p* = 0.027; Table 1; Figure 4). The post hoc contrast tests revealed that SD‐ankle‐torque was significantly lower in Unpleasant than in Pleasant (*p* = 0.026; Table 2; Figure 4). In contrast, no significant main effect or interaction was found in SD‐knee‐torque or SD‐hip‐torque found (All *p* > 0.05; Table 1; Figure 4).

### 3.5 Breathing variables

**FIGURE 5:**
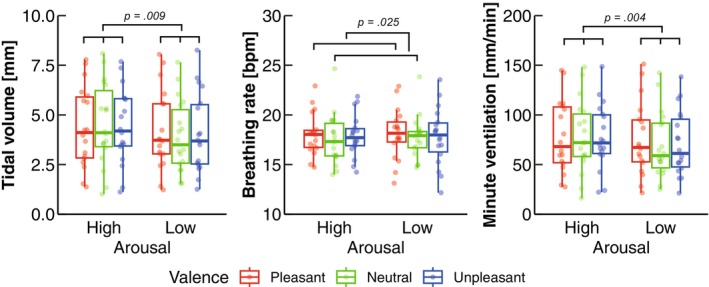
Group data for the breathing variables. The lines in the box plots indicate median values, and the ends of the boxes represent the 25th and 75th percentiles. The dots represent individual data points. The dots represent individual data points. The significant *p* values (*p* < 0.05) are indicated.

The two‐way ART‐ANOVA showed main effects of arousal on the tidal volume (*p* = 0.009) and the minute ventilation (*p* = 0.004; Table 1; Figure 5), indicating higher values in High than in Low (Table 2; Figure 5). In contrast, the two‐way ART‐ANOVA revealed a significant main effect of valence on the breathing rate (*p* = 0.029; Table 1; Figure 5). The post hoc test indicated higher value in Pleasant than in Neutral (*p* = 0.025; Table 2; Figure 5).

### 3.6 Correlation analysis

Since SD‐COM was significantly lower in Unpleasant than in Neutral and Pleasant (see Section 3.4), we calculated the change ratio of each parameter as follows:

(6) $$\text{Change ratio}\;\left[\%\right]=\frac{M_{\text{Unpleasant}}-M_{\text{Pleasant or Neutral}}}{M_{\text{Pleasant or Neutral}}\;}$$

Where $M_{\text{Unpleasant}}$ is the value in Unpleasant and $M_{\text{Pleasant or Neutral}}$ is the value in Pleasant or Neutral. Each value was calculated by averaging High and Low arousal levels. Subsequently, the change ratio was used to calculate correlation coefficient between variables.

**FIGURE 6:**
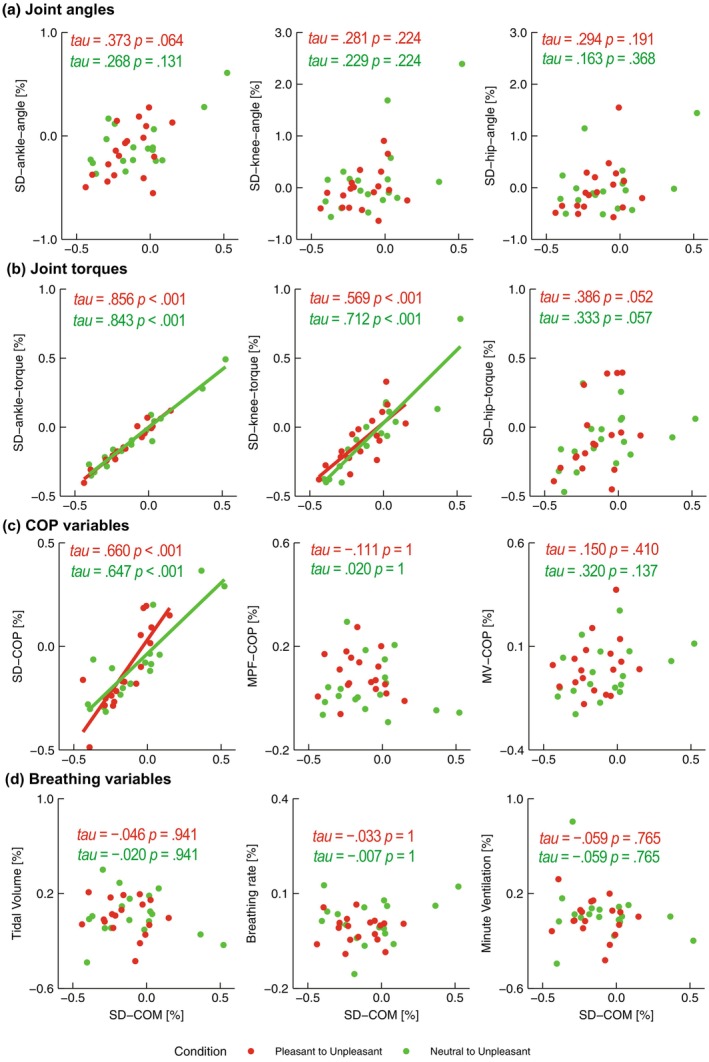
Scatter plots showing the relationships between SD‐COM and other standing kinematic and kinetic variables (a. Joint angles; b. Joint torques; c. COP variables; d. Breathing variables). Red dots represent change ratios from Pleasant to Unpleasant, while green dots indicate change ratios from Neutral to Unpleasant. Regression lines are shown for correlations that were statistically significant (*p* < 0.05).

Figure 6 shows the correlation relationships between SD‐COM and other kinematic and kinetic parameters. As for joint angles, no significant correlation was found between SD‐COM and any of the joints (Figure 6a). Among the joint torques, significant positive correlations with SD‐COM reduction from Pleasant/Neutral to Unpleasant were observed for the ankle and knee, but not for the hip (Figure 6b). For COP variables, the reduction in SD‐COM from Pleasant/Neutral to Unpleasant was also significantly positively correlated with SD‐COP, but not with MPF‐COP or MV‐COP (Figure 6c). No significant correlations were found between SD‐COM and breathing variables (Figure 6d).

## 4 DISCUSSION

In the present study, we aimed to investigate the effects of arousal and valence on body kinematics and kinetics during quiet standing using a motion capture system. Regarding the COP variables, our results showed more frequent COP sway (MPF‐COP) in unpleasant emotions compared to pleasant emotions (Figure 3), which is consistent with previous reports (Azevedo et al., 2005; Facchinetti et al., 2006; Takahashi, Kaneko, Yokoyama, et al., 2024). While previous studies have focused on COP responses, our findings offer a novel perspective by examining the body kinematics and kinetics. Specifically, we identified valence effects on postural kinematics and kinetics, indicating that unpleasant emotions reduced variability in the COM, ankle angle, and ankle torque, but not in the hip or knee (Figure 4), partially supporting H‐1. Furthermore, as anticipated, high arousal emotions increased minute ventilation—a parameter not previously explored in similar contexts—supporting our second hypothesis (H‐2) (Figure 5). In addition to this, high arousal emotions increased tidal volume, while pleasant emotions led to a higher breathing rate compared to neutral emotions. Besides, the significant effect of arousal on tidal volume and of valence on heart rate objectively demonstrates the successful manipulation of arousal and valence levels (Figures 2 and 5). These results are further discussed in the following sections.

### 4.1 Emotional effects on postural kinematics and kinetics

We firstly showed that COM variability (SD‐COM) was reduced in unpleasant emotions compared to neutral and pleasant emotions, indicating a reduction in postural sway under unpleasant emotions. Considering the similarity between COM and COP trajectories (Winter et al., 1998), COP variability (SD‐COP) may also decrease under unpleasant emotional states, as observed for COM variability. While the reduction in COM variability under unpleasant emotions was significantly correlated with that in COP variability (Figure 6c), which preserved a trait normally observed during quiet standing (Masani et al., 2014), COP variability was not significantly different across emotional conditions, which contradicts our previous report (Takahashi, Kaneko, Yokoyama, et al., 2024). This discrepancy between COM and COP in the present study would be attributed to the fact that COP and COM reflect fundamentally distinct physiological processes in postural control: COM directly represents body sway, whereas COP is almost proportional to ankle torque to control COM (Winter et al., 1998). Although the reason for the discrepancy in SD‐COP between the present study and our previous work (Takahashi, Kaneko, Yokoyama, et al., 2024) remains unclear, this discrepancy may be attributed to differences in the degree of subjective valence between the two studies. We calculated the effect size (*d*) for the difference in subjective valence ratings—assessed with the same SAM scale in both studies—between the two valence levels (Pleasant vs. Unpleasant). The present study showed a smaller change in SAM scale compared with the previous study (present study: *d* = 4.93; previous study: *d* = 5.62), possibly yielding the significant valence effect on SD‐COP only in the previous study.

Regarding joint angles and torques, our results showed a significant reduction in the variability of ankle angle and torque under unpleasant emotional conditions, whereas no such reduction was observed for the hip or knee. During quiet standing, each of ankle and hip joint torques contributes to reducing the net angular acceleration of the other joint, thereby minimizing the COM acceleration (Sasagawa et al., 2014), indicating that quiet standing is regulated through inter‐joint coordination (Shanbhag et al., 2023). Regarding this, recent simulation studies revealed that the central nervous system (CNS) does not encode this inter‐joint coordination (Morasso, 2022; Morasso et al., 2019). Specifically, hip torque is generated by intrinsic mechanical properties (e.g., stiffness and viscosity), and only a slight amount of muscle tonus from the CNS is required to enhance these properties. Their findings are also supported by a study that revealed the lack of coherence between trunk movement and trunk muscular activities (Saffer et al., 2008). In contrast, the generation of ankle torque requires not only stronger muscle tonus to support intrinsic mechanical properties than is needed for hip torque, but also phasic muscle activity driven by sensory feedback related to ongoing sway. Accordingly, the CNS preferentially controls ankle torque during quiet standing, while ankle–hip coordination merely reflects an implicit biomechanical consequence (Morasso, 2022; Morasso et al., 2019). Taken together with the previous studies, our results—showing a significant reduction in the variability of ankle angle and torque under unpleasant emotional conditions—suggest that the CNS relies primarily on ankle‐driven postural control in response to unpleasant stimuli.

Then, is the reduction in variability of ankle angle and torque under unpleasant emotions associated with the reduction in COM variability (i.e., postural sway)? There was no significant correlation between ankle angle and COM variability (Figure 6a), whereas ankle torque variability showed a significant correlation with COM variability (Figure 6b). These results are in line with previous studies reporting that COM correlates more strongly with ankle torque than ankle angle (Ghazaleh et al., 2014). Given the fact that COM reflects the combined contributions of the ankle, knee, and hip joint angles, antiphase relationships among these joints may reduce COM variability, regardless of the variability in each individual joint angle (Creath et al., 2005). This could explain the absence of a significant correlation between COM variability and ankle angle variability. In contrast, given that the EMG activity of the triceps surae muscles is synchronized with COM, it is reasonable that the variability of ankle torque—directly influenced by muscle activity—is associated with COM variability (Masani et al., 2003). Furthermore, the gastrocnemius muscle, as a biarticular muscle, contributes not only to ankle torque but also to knee torque. This supports the present finding that variability in knee torque was also correlated with COM variability (Figure 6b), consistent with previous observations (Günther et al., 2009). That is, the CNS may actively modulate ankle torque to control postural sway, with knee torque playing a supportive role under unpleasant emotional conditions.

Another interest is what caused the reduction in variabilities of the COM, ankle angle, and ankle torque in unpleasant emotions. Although abdominal breathing movements are major factors of postural sway (Bouisset & Duchêne, 1994; Hodges et al., 2002), variability of the abdominal displacement (i.e., the tidal volume) was affected by arousal but not valence (Figure 5) and was not significantly correlated with reduction in COM variability under unpleasant emotions (Figure 6d). These findings suggest that abdominal movement would not be related to the postural kinematic and kinetic changes in unpleasant emotions. Alternatively, these changes may be attributed to unpleasant emotions modulating the CNS for postural control. Recent evidence suggests that the CNS activates postural muscles intermittently during quiet standing based on sensory feedback, as a strategy to minimize energy expenditure (Asai et al., 2013; Zhao, Hodossy, et al., 2024; Zhao, Zhang, et al., 2024). When this intermittent control shifts toward a continuous mode, postural sway (e.g., COM variability) tends to decrease, reflecting more frequent and precise adjustments by the CNS. Accordingly, the observed reduction in COM variability under unpleasant emotional conditions may be attributed to a transition from intermittent to continuous postural control. This interpretation is supported by reports that unpleasant emotions enhance somatosensory input from the ankle muscle spindles (Ackerley et al., 2017) and precise perception of ankle movement (Samain‐Aupic et al., 2019). Taken together, unpleasant emotions might have enhanced sensory feedback, allowing adjustments in ankle torque to approach the equilibrium point (i.e., reduced variability of ankle torque), leading to reduced variability in the ankle angle and COM. In the future, it will be necessary to clarify the detailed neural mechanisms underlying postural kinematic and kinetic changes associated with unpleasant emotions.

### 4.2 Abdominal breathing movements are key emotional parameters during quiet standing

Given the existence of emotional effects on abdominal breathing, which is a detectable movement in the standing kinematics (Boiten, 1998; Gomez et al., 2004, 2005, 2008; Gomez & Danuser, 2004; Gomez, Filippou, et al., 2016), we focused on the effects of arousal and valence on breathing motion. In line with our expectation, the minute ventilation was significantly increased in high arousal emotions compared to low arousal emotions. Many psychophysiological studies have provided consistent evidence that the minute ventilation is a sensitive breathing variable to arousal states (Boiten, 1998; Gomez et al., 2004, 2005, 2008; Gomez & Danuser, 2004; Gomez, Filippou, et al., 2016). Additionally, it has been indicated that increased minute ventilation in high arousal emotions comes from increased tidal volume but not changes in breathing rate (Gomez et al., 2008), covering the increased tidal volume under high arousal states in the present study. Moreover, we observed valence effects on breathing rate, with higher breathing rates in pleasant emotions compared to neutral, but not compared to unpleasant emotions. This suggests that the influence of valence on breathing rate may not follow a simple linear trend. Gomez et al. (2008) reported a linear valence effect, demonstrating that more pleasantness were associated with increased breathing rates when viewing IAPS images in sitting posture. Therefore, the modulation of breathing rate in response to emotional images may be posture‐dependent. Specifically, since the breathing rate is generally higher in standing posture than in sitting posture, a reduction in breathing rate in response to unpleasant stimuli observed during sitting might be less detectable in standing posture. In addition, the effects of arousal and valence on most breathing variables are very complex, and results are influenced by stimulus type (e.g., picture, film, or music) (Gomez et al., 2004, 2005, 2008; Gomez & Danuser, 2004; Gomez, Filippou, et al., 2016). Nevertheless, the minute ventilation is well established as a reliable parameter of arousal level regardless of stimulus type (Boiten, 1998; Gomez et al., 2004, 2005, 2008; Gomez & Danuser, 2004; Gomez, Filippou, et al., 2016), and therefore the present study stresses the usefulness of breathing kinematics during quiet standing for arousal detection.

### 4.3 Practical implications

Recent advances in machine learning and artificial intelligence (AI) have led to the development of systems for estimating emotional states, with physiological (e.g., electroencephalography) or behavioral (e.g., kinematics) parameters, thereby enabling the monitoring of mental health status in both healthy individuals and clinical populations, including those with psychiatric disorders. Regarding behavioral parameters, most studies have primarily focused on dynamic movements such as gait (Deligianni et al., 2019). In contrast, there remains significant potential for further exploration in emotional detection using standing parameters (Riemer et al., 2023). The present study demonstrated that kinematic and kinetic parameters in quiet standing measured by motion capture are sensitive to emotional states, suggesting that these standing parameters may serve as viable modalities for emotional recognition. Standing posture offers a distinct advantage as an experimental procedure: it is static and does not involve dynamic movements, which allows for the combination of behavioral and physiological signals, such as electroencephalography, with minimal movement artifacts. This could lead to more precise emotional recognition. Furthermore, recent developments in marker‐less motion capture systems suggest that emotion estimation using standing parameters could be achieved in a practical manner (Avogaro et al., 2023).

### 4.4 Limitations

First, while the present study showed a valence effect on COP frequency, our previous study demonstrated an arousal effect on it (Takahashi et al., 2025). Given that both studies confirmed successful manipulations of arousal and valence through subjective (rating) and objective (autonomic nervous activity) indicators, it is likely that both factors influence postural control. The discrepancy between the two studies may be attributable to differences in the magnitude of arousal and valence changes elicited across conditions. We calculated the effect size (*d*) for the difference in subjective arousal ratings (i.e., SAM) between the two arousal levels (High vs. Low) and in subjective valence ratings between the two valence levels (Pleasant vs. Unpleasant). The present study showed a greater change in valence (present study: *d* = 4.93; previous study: *d* = 3.45) and a smaller change in arousal compared with the previous study (present study: *d* = 1.11; previous study: *d* = 2.03). Therefore, while both arousal and valence can influence postural control, they may diminish each other's effects, or such effects might only become apparent when emotional changes exceed a certain threshold. In the present study, there was some overlap in picture content across conditions, which may have reduced the arousal effect size to about half that of the previous study (Bradley et al., 2001; Lang & Bradley, 2010).

Second, we modeled the upper body as one segment (i.e., HAT). For quiet standing, lower limb joints play the most significant roles in postural control, and therefore it is general to use the HAT segment (Sasagawa et al., 2009; Yamamoto et al., 2015). On the other hand, it has also been indicated that the six joints, where the HAT segment is decomposed into neck, elbow, and hand joints, coordinate with each other to stabilize the COM and head positions even in quiet standing (Wu et al., 2009). This report suggests that quiet standing is controlled with whole‐body coordination. In the future, it seems better to focus on the emotional effects on upper body kinematics and kinetics during quiet standing by modeling the upper body as a multi‐segment structure.

Third, the present study recruited only male participants. Interoception, the process of monitoring internal bodily states such as organ activity (Craig, 2002), is known to be associated with emotional responses and body posture (Dohata et al., 2025; Prentice et al., 2022). To eliminate the potential effects of the menstrual cycle on interoception in female participants, only male participants were selectively recruited. However, given reported gender differences in physiological responses to affective pictures (Gomez, von Gunten, & Danuser, 2016), caution is warranted in generalizing the present findings beyond healthy male participants. Future studies should include female participants to examine potential gender differences.

Finally, while this study defined breathing movement based on abdominal displacement, it should be noted that thoracic movement is also an integral component of respiration. Given the individual differences in abdominal and thoracic breathing patterns, abdominal breathing movements may not fully capture emotional modulation. Moreover, the abdominal displacement was quantified using a two‐dimensional distance. From a strictly kinematic perspective, the estimation of respiratory parameters ideally involves three‐dimensional measurements encompassing both thoracic and abdominal regions (Romei et al., 2010). Therefore, although the current method provides a simple and practical approach to evaluating breathing characteristics, it may not fully capture the complexity of respiratory mechanics. In particular, because tidal volume is inherently a volumetric measure (e.g., liter), future studies may benefit from more comprehensive measurement systems incorporating additional markers and three‐dimensional reconstruction for increased accuracy.

## 5 CONCLUSION

Our results indicate that body kinematics and kinetics during quiet standing reflect both arousal and valence states. Specifically, postural parameters are sensitive to valence states, while abdominal breathing movements are indicative of both arousal (e.g., tidal volume and minute ventilation) and valence (e.g., breathing rate). These findings enhance our fundamental understanding of the emotional impact on behavioral responses, suggesting that quiet standing parameters detected by a motion capture system can be a valuable tool for emotional evaluation.

## AUTHOR CONTRIBUTIONS

RT contributed to conceptualization, data curation, formal analysis, investigation, funding acquisition, methodology, visualization, and writing—original draft. NK contributed to data curation, supervision, methodology, and writing—review and draft. KN contributed to conceptualization, supervision, funding acquisition, and writing—review and draft. All authors contributed to the article and approved the submitted version.

## FUNDING INFORMATION

This work was supported by Japan Society for the Promotion of Science (JSPS) for Fellows Grant‐in‐Aid (KAKENHI) to RT [#24KJ0729], the JST‐SPRING to RT [#JPMJSP2018], and the JST‐MOONSHOT program to KN [#JPMJMS2012‐2‐2‐2].

## CONFLICT OF INTEREST STATEMENT

The authors declare that the research was conducted in the absence of any commercial or financial relationships that could be construed as a potential conflict of interest.

## ETHICS STATEMENT

The study was conducted in accordance with the Declaration of Helsinki and approved by the local Ethics Committee of the University of Tokyo [#792]. The participants provided their written informed consent to participate in this study.

## Data Availability

The data presented in this manuscript were newly acquired for the present study. Because of data privacy concerns, they are not available to the community in open repositories. The datasets generated in this present study are available from the corresponding author upon reasonable request. In such cases, the reason for the data request and procedures for ensuring privacy will be reviewed and discussed.

## Appendix

(1) High‐Pleasant: 4150, 4220, 4275, 4279, 4608, 4611, 4676, 4690, 8180, 8200, 8370, 8470; (2) High‐Neutral: 1040, 1070, 1110, 1114, 1120, 1300, 1321, 1931, 1932, 5940, 5971, 5972; (3) High‐Unpleasant: 2811, 3030, 3068, 3069, 3110, 3400, 6250, 6313, 6540, 9250, 9252, 9635; (4) Low‐Pleasant: 1610, 1920, 2040, 2058, 2071, 2080, 2154, 2332, 2540, 2550, 5594, 5814; (5) Low‐Neutral: 2446, 2695, 2752, 2810, 5970, 7046, 7054, 7360, 7487, 9404, 9472, 9913; (6) Low‐Unpleasant: 2141, 2750, 3017, 3230, 3301, 9000, 9301, 9340, 9433, 9435, 9830, 9900.
